# Development and Characterization of Somatic Hybrids of *Ulva reticulata* Forsskål (×) *Monostroma oxyspermum* (Kutz.)Doty

**DOI:** 10.3389/fpls.2015.00003

**Published:** 2015-01-29

**Authors:** Vishal Gupta, Puja Kumari, CRK Reddy

**Affiliations:** Seaweed Biology and Cultivation Group, Discipline of Marine Biotechnology and Ecology, CSIR-Central Salt and Marine Chemicals Research InstituteBhavnagar, India

**Keywords:** heterosis, hybrid, *Monostroma*, protoplast, *Ulva*

## Abstract

Ulvophycean species with diverse trait characteristics provide an opportunity to create novel allelic recombinant variants. The present study reports the development of seaweed variants with improved agronomic traits through protoplast fusion between *Monostroma oxyspermum* (Kutz.) Doty and *Ulva reticulata* Forsskål. A total of 12 putative hybrids were screened based on the variations in morphology and total DNA content over the fusion partners. DNA-fingerprinting by inter simple sequence repeat (ISSR) and amplified fragment length polymorphism (AFLP) analysis confirmed genomic introgression in the hybrids. The DNA fingerprint revealed sharing of parental alleles in regenerated hybrids and a few alleles that were unique to hybrids. The epigenetic variations in hybrids estimated in terms of DNA methylation polymorphism also revealed sharing of methylation loci with both the fusion partners. The functional trait analysis for growth showed a hybrid with heterotic trait (DGR% = 36.7 ± 1.55%) over the fusion partners *U*. *reticulata* (33.2 ± 2.6%) and *M*. *oxyspermum* (17.8 ± 1.77%), while others were superior to the mid-parental value (25.2 ± 2.2%) (*p* < 0.05). The fatty acid (FA) analysis of hybrids showed notable variations over fusion partners. Most hybrids showed increased polyunsaturated FAs (PUFAs) compared to saturated FAs (SFAs) and mainly includes the nutritionally important linoleic acid, α-linolenic acid, oleic acid, stearidonic acid, and docosahexaenoic acid. The other differences observed include superior cellulose content and antioxidative potential in hybrids over fusion partners. The hybrid varieties with superior traits developed in this study unequivocally demonstrate the significance of protoplast fusion technique in developing improved varients of macroalgae.

## Introduction

Marine macroalgae (seaweeds) consist of an assemblage of genetically, morphologically and functionally diverse photoautotrophic species that carry out key ecosystem functions in the marine environment. Seaweeds are historically consumed as human food in several Asian Countries and as raw material for extraction of industrially import phycocolloids (agar, carrageenan and alginate) and recently as phycosupplements, nutraceutical, cosmetics, bioactive substances etc. (Holdt and Kraan, 2011). The beneficial utilization of seaweed resources for humankind has provided impetus for domestication of economically important seaweeds leading to their commercial cultivation. There has been a substantial growth in the production of seaweeds from 3.8 million fresh tons per annum in 1998 to about 25 million fresh tons in 2012 (FAO, 2014). A significant proportion of seaweed produced, representing about 76% of the tonnage of total harvest (88% of the value of entire seaweed industry) is consumed as human food or animal feed (FAO, 2012). The emergence of seaweed aquaculture as a promising seaweed industry has opened up avenues for developing varieties with agronomically improved traits for higher economic gains. However, genetic improvement programmes are seriously lacking in seaweeds and conventional practices were largely resorted to selection of clones with useful traits from wild populations followed by development of homogenous lines (Robinson et al., 2013). The continuous rigorous selection from the same progeny on long run may deplete genetic diversity causing a gradual decline in adaptability to changing environments and eventually affecting the vigor of economic traits.

Selective breeding, though applied for developing elite varieties, is largely restricted to a few seaweed genera such as *Porphyra* and *Laminaria*. For example, only 30 cultivars of *Porphyra* have so far been developed ever since its life cycle and control of reproduction became known in 1949 (Robinson et al., 2013). Similarly, a few cultivars of *Laminaria* such as Danhai No. 1 (Ou et al., 1983), Danza No. 10 (Fang et al., 1985), Danhai No. 901 (Zhang et al., 2001), and recently the Dongfang No. 2 (Li et al., 2007) have been developed since the first gametophyte cloning and gametophyte clone hybridizing methods were developed in 1970 (Fang et al., 1978; Zhou and Wu, 1998). Nevertheless, selective breeding has remained inconclusive for other seaweed groups such as agarophytes, carragenophytes and green seaweeds. Attempts to cross different *Gracilaria* species (Bird and McLachlan, 1982; Plastino and De Oliveira, 1988) and *Ulva* species (Hiraoka et al., 2003, 2011) failed due to sexual incompatibility barriers.

Somatic hybridization through protoplast fusion is a useful technique that facilitates recombination of economically important traits by harnessing the natural genetic diversity from phylogenetically distant species (Hodge et al., 2010). This technique has been extensively applied in terrestrial plants and showed unprecedented success in developing numerous agricultural varieties improved for either productivity or resistance to biotic/abiotic factors (Davey et al., 2005; Li et al., 2014; review Liu and Xia, 2014). However, this area of research for seaweeds lags far behind that of land plants despite considerable success in protoplast isolation and regeneration in numerous seaweed species (reviewed in Reddy et al., 2008). In the past, a few attempts have been made to develop somatic hybrids of *Porphyra* but most failed to regenerate hybrid plants. Nevertheless, a few succeeded in developing chimeric thalli with no significant benefits. Most importantly, these studies lacked the characterization of regenerated hybrids at a genetic level to confirm the genetic recombination (reviewed in Reddy et al., 2008). Thus, this field remained largely inconclusive to date.

This study aimed at development of hybrid varieties with improved functional traits through intergeneric protoplast fusion between *Monostroma oxyspermum* (Kutz.) Doty and *Ulva reticulata* Forsskål. These Ulvophycean species are cultivated at a commercial scale in East Asian countries for utilization in various food preparations. In recent times, western countries have also accepted the utility of these species in seafood cuisines (Mouritsen et al., 2013). Most recently, the genus *Ulva* has also been strongly advocated as potential source of non-lignocellulosic feedstock for production of renewable fuels along with other associated high value chemicals (Trivedi et al., 2013). Therefore, it is of immense interest to highly desirable to generate genetic variants of Ulvophycean species by harnessing the prevalent vast natural genetic diversity.

## Materials and methods

### Selection of seaweed species

The species *Monostroma oxyspermum* (Kützing) Doty and *Ulva reticulata* Forsskål were selected for development of somatic hybrids through protoplast fusion. Henceforth, these two species are mentioned as “fusion partners” in this manuscript. These species were selected based on the higher growth rate of *U*. *reticulata* and nutritional values of *M*. *oxyspermum*. The thalli of *M*. *oxyspermum* were collected from mangrove swamps at Achra, central west coast of India (16° 11′N, 73° 26′E) and *U*. *reticulata* was collected from Kalubhar Island, north western coast of India (22° 29′N, 69° 37′E). The thalli were brought to the laboratory under cool conditions in a thermos flask. Young thalli of each species were cleaned thoroughly with autoclaved seawater for removal of dirt and adhering particles. The cleaned material of each species was maintained in unialgal culture in the laboratory. The unialgal culture was established by growing the algal thalli in sterile enriched seawater media (Provasoli, 1968) supplemented with GeO_2_(10 mgL^−1^) for a week under white fluorescent lamps of irradiance intensity about 15 μmole photon m^−2^ s^−1^ with a 12: 12 h light:dark photoperiod. During this period, the culture media was changed every 2 days.

### Protoplast isolation and fusion

Axenic culture of algal thalli for both the fusion partner species and subsequent protoplast isolation from them was carried out following the protocol described by Krishnakumar et al. (1999) and Reddy et al. (2006). Protoplasts from both species were suspended in seawater in a 1:1 ratio (1.0 × 10^5^ cellsml^−1^) and were washed twice with electro-fusion buffer [0.8 M mannitol, 0.2 mM tris (hydroxymethyl) amino-methane, 1 mM CaCl_2_ and 1 mM MgCI_2_ in distilled water, adjusted to pH 7.5]. An aliquot of 100 μl protoplast mix was placed between the two electrodes (1 mm spacing) in a fusion chamber (FTC-2, Shimadzu Co, Japan) and allowed to settle for few minutes prior to the start of the electrofusion. The electric field-mediated protoplast fusion was performed using a Somatic hybridizer SSH-2 (Shimadzu Co., Japan). Protoplasts were initially aligned into short chains, preferably pairs of 2 to4 protoplasts in an alternating current (AC) field and subsequently fused by the application of a single short-duration direct current (DC) pulse. The alignment of the protoplasts on application of AC voltage was optimized by varying the voltage from 10 to 40 V for different durations, from 10 to 25 s. The fusion frequency was also optimized by determining the optimum DC pulse voltage and pulse width. For this, the DC pulse voltage was varied from 100 to 350 V and pulse width was varied from 10 to 60 μs.

### Culture of heteroplasmic fusion products

The heteroplasmic fusion products were identified based on the presence of two morphologically distinct chloroplasts in a single cell and the larger size of the cell compared to unfused protoplasts. Presumptive hybrid products were immediately transferred to a separate Petri-dish using glass pipette and cultured in seawater containing 0.4 M mannitol in dim light of irradiance 10 μmol photon m^−2^ s^−1^ using day light fluorescent lamps with 12:12 L:D photoperiod at 20 ± 1°C for 2 days and later transferred to increased irradiance of 25 μmol photon m^−2^ s^−1^. After settlement of presumptive hybrid products, the medium was replaced with sterile seawater. The cellular division and regeneration was monitored at regular time intervals. After 15 days of culture, when presumptive hybrids had developed to many-celled stages, seawater was supplemented with PES medium (Provasoli, 1968). After attainment of thalli around 1 cm in length, plantlets were transferred to round flat bottom aerated flasks and cultured under the same conditions. The culture medium was replenished frequently to sustain cell division and growth.

### Characterization of the fusion products

The fusion products obtained were first screened based on their morphological distinctiveness compared to fusion partners, followed by ploidy analysis and molecular level characterization.

### Ploidy analysis using confocal microscopy

Thalli of each regenerant were sectioned and processed for ploidy analysis using confocal microscopy (Shimizu et al., 2008; Zitta et al., 2012). Briefly, the sections of thalli were incubated with 4,6-diamidino-2-phenylindole (DAPI) at a concentration of 0.5 μg mL^−1^ in sea water for 1 h (Sheahan et al., 2004). The material was mounted on slides and observed with a confocal microscope (Carl Zeiss). DAPI-stained nuclei were observed at 405 nm (blue) laser wavelength excitation with emission spectrum from 510 to 566 nm. Chloroplast auto- fluorescence was observed at 488 nm (violet) laser wavelength excitation with emission spectrum from 639 to 701 nm. Bright-field images were also captured, as a reference.

Images of nuclear fluorescence were digitized and reduced to 300 kB with 8 bits to facilitate the quantification of fluorescent dots. Images were captured in only one plane to avoid duplication of nuclear fluorescence. The intensity of pixels in these areas was calculated with the inbuilt software (Carl Zeiss). For each genotype, 100 nuclear areas were randomly selected and analyzed. Among the fusion partners, the one that showed the highest fluorescence intensity was defined as 100% and fluorescence intensity for rest of the genotypes was estimated relative to this.

### Molecular characterization

The molecular characterization of putative hybrids included multi-locus genotyping by inter simple sequence repeat (ISSR), and amplified fragment length polymorphism (AFLP) markers. In addition to these markers, epigenetic variations among the parental genotypes and their transmission to hybrid progeny was determined by a methylation sensitive amplification polymorphism (MSAP) assay.

### Genomic DNA extraction

Genomic DNA from the putative hybrids and parental thalliwas extracted by modified cetyltrimethyl ammonium bromide (CTAB) method. Thalli were blotted with tissue paper, homogenized in liquid nitrogen and mixed with preheated (65°C) CTAB buffer consisting of [2% CTAB (w/v), 1.4 M NaCl, 100 mM Tris–HCl (pH 8.0), 50 mM ethylene diamine tetra acetate (EDTA 2Na), 50 mM sodium sulphite and 1% PVP] and incubated at 65°C in a water bath for 1 h with occasional gentle mixing. The cooled mixture was extracted twice with an equal volume of chloroform: isoamyl alcohol (24:1), centrifuged at 12,000 × g for 5 min and the upper aqueous layer was collected. The recovered aqueous layer was treated with RNase A (10 μg) for 30 min at 37°C. DNA was precipitated with isopropanol and pelleted by centrifugation at 12,000 × g. The DNA pellet was washed with a mixture of ethanol (76%) and sodium acetate (0.2 M). A second wash was performed with mixture of pure ethanol and 10 mM ammonium acetate and the resulting pellet was air dried and finally rinsed with pure ethanol. The final DNA pellet obtained was dissolved in Tris-EDTA (10 mM, pH 8.0). The purity of the extracted DNA was checked by measuring the OD_260_/OD_280_ ratio and by agarose gel electrophoresis.

### ISSR analysis

A total of 20 ISSR primers were screened for amplification of which 8 gave a highly reproducible band profile (Supplementary Table 1). Amplification was carried out in a Bio-Rad thermal cycler (USA). The ISSR programming involved an initial denaturation step of 3 min at 94°C followed by 45 cycles at 94°C for 40 s, respective primer annealing temperature for 45 s and 72°C for 1.5 min, with final extension step at 72°C for 5 min. The amplicons were size fractionated by electrophoresis on 3% agarose gel and bands were visualized under UV light after ethidium bromide staining.

### AFLP analysis

AFLP analysis was carried out using a Life Technologies (USA) commercial kit following the instructions given in the manual supplied with the kit. Purified genomic DNA from the parent and the putative hybrids were restriction digested with *EcoR*I and *Mse*I endonucleases for 5–6 h at 37°C followed by denaturation for 1 h at 65°C. Two adapters were then ligated to the sticky ends of the digested DNA. The ligation reaction was carried out for 16–18 h at 20°C. The resultant ligated products were diluted and were pre-amplified using adapter-specific primers. Resulting PCR products were diluted and were amplified using 12 pairs of randomly selected primers from the commercial kit (Life Technologies, USA). The final PCR products were visualized on 6% polyacrylamide gel by silver staining (Bassam et al., 1991).

### ISSR and AFLP data analysis

Both these experiments were carried out in duplicate to ensure for assuring the consistency of the bands. The fractionated ISSR and AFLP profiles were scored for each genotype as discrete characters based on the presence or absence of amplified bands (Ruas et al., 2003; Toppino et al., 2008). Each locus was then analyzed for the presence or absence of the band. The band profile was converted into a binary matrix of 1 or 0 depending on the presence and absence of band respectively. The frequency of shared and polymorphic bands between hybrids and fusion partners were then determined.

### MSAP analysis

MSAP analysis was performed according to the method described by Gupta et al. (2012). Briefly, genomic DNA from each thallus type was cleaved with *Eco*RI and either *Hpa*II or *Msp*I restriction endonucleases. The restricted fragments were ligated with *Eco*RI and *Hpa*II/*Msp*I adapters (Supplementary Table 2) using T4 DNA ligase. Primary PCR amplification was carried out using primers complementary to the *Eco*RI and *Hpa*II/*Msp*I adapters with one additional selective nucleotide at the 3′ end (Supplementary Table 3) where ligated DNA fragments served as templates. PCR products thus obtained were diluted and used as templates for secondary selective amplification with combinations of primers complementary to the *Eco*RI and one of the *Hpa*II/*Msp*I adaptors, but this time with two or three selective nucleotides, respectively, at the 3′ end (Supplementary Table 4). The final PCR products were visualized on 6% polyacrylamide gel by silver staining (Bassam et al., 1991).

### MSAP data analysis

MSAP data originated from the electrophoresis of PCR products were converted into a binary matrix of 1 and 0 based on the presence or absence of bands. An amplification pattern of the type 11 corresponded to samples displaying bands in both the *Msp*I and *Hpa*II lanes and was defined as an unmethylated site. Amplification patterns of the type 01 corresponded to samples showing an amplified band after restriction with *Msp*I but not after restriction with *Hpa*II (internal cytosine fully-methylated site). Pattern 10 corresponded to samples displaying an amplified band after restriction with *Hpa*II but not after restriction with *Msp*I (external cytosine hemi-methylated site). Pattern 00 indicated no band amplified after restriction with either isoschizomer, revealing either full methylation at the locus in both cytosines, or full-methylation of the external cytosine. Two replicates of DNA extraction were performed from two different sets of cultured protoplasts. MSAP was also performed in duplicate to assess the reproducibility and consistency of amplification profile.

All the aforementioned analyses were carried out in replicates of two to confirm the reproducibility of the results. The distribution matrix for all genotypes, i.e., parent and putative hybrids based on all the analyses was analyzed using the software package *msap*ver 1.1.0 (Pérez-Figueroa, 2013).

### Functional traits analysis

The traits analyzed for characterization of the putative hybrids were: growth rate, thermal tolerance and proximate composition analysis including lipid/fatty acid composition, total protein content, total carbohydrate, cellulose content and C, H, and N analysis. Before analysing these functional traits, seaweed thalli were mass propagated by vegetative fragmentation in sterile seawater supplemented with PES medium enrichment. The biomass thereby obtained was made axenic and cultured for 1 week before analyzing these traits.

### Determination of growth rate

The growth rate of selected genotypes was determined as a measure of daily growth rate or DGR (%). For the DGR (%) calculation, thalli of each genotype weighing 100 mg were cultured in 500 mL round flat bottom aerated flasks containing 400 mL sterile seawater supplemented with PES medium for 15 days at temperature 25°C under white cool fluorescent light of irradiance 50 μmol photons m^−2^ s^−1^ under a day-night photoperiod of light: dark 12: 12 h. During this culture duration the medium was changed every third day. The DGR (%) was measured for each genotype by estimating the weight of each thallus at every third day. The DGR (%) was calculated according to the equation DGR (%) = (W_t_/W_0_)^1/t^ - 1 × 100 where W_t_ is the weight after time t and W_0_ is the initial weight at time t = 0.

### Proximate composition analysis

The proximate composition including protein, carbohydrateand lipid content was determined following the methods of Lowry et al. (1951), Dubois et al. (1956) and Bligh and Dyer (1959). Cellulose profiling was carried out following the method described by Siddhanta et al. (2009). For lipid extraction, thalli were first washed with distilled water to remove surface salts and then blotted with paper towels. Lipids were extracted following the method of Bligh and Dyer (1959). The samples were ground to a fine powder in liquid N_2_ and extracted with chloroform: methanol (1: 2, v/v). The residue obtained was re-extracted three times with small amount of chloroform: methanol (1: 1, v/v). Extracts were filtered and mixed with chloroform and water for phase separation. The organic phase was collected and evaporated to dryness *in vacuo* to obtain total lipids. For fatty acid profiling, fatty acids from lipids were converted into their respective methyl esters by trans-methylation using 1% NaOH in methanol and heated for 15 min at 55°C, followed by the addition of 5% methanolicHCl and again heated for 15 min at 55°C. Fatty acid methyl esters (FAMEs) were extracted in hexane and analyzed by GC-2010 coupled with GCMS-QP2010. FAMEs peaks were identified by comparing their retention time with those of the standard mixture (FAME mix C4-C24) by GCMS post-run analysis and quantified by area normalization.

The total content of phenolic compounds was determined spectrophotometrically using Folin-Ciocalteu reagent following Lim et al. (2002). In brief, extracts at a concentration of 1 mg mL^−1^ were first diluted with methanol, and then an aliquot of 0.1 mL from the diluted extracts was added into the test tubes. To this, 2.9 mL of distilled water and 0.5 mL of Folin-Ciocalteu's reagent were added and mixed thoroughly. After 10 min, 1.5 mL of 20% sodium carbonate solution was added, and the mixture was mixed thoroughly and allowed to stand at room temperature in the dark for 1 h. Absorbance was measured at 725 nm, and the total content of phenolic compounds was calculated based on a standard curve of phloroglucinol.

Total antioxidant activity (TAC) was determined according to Prieto et al. (1999). In brief, the methanolic extract at concentration of 1 mg mL^−1^ was mixed with 3.0 mL of reagent solution containing 0.6 M H_2_SO_4_, 28 mM sodium phosphate, and 4 mM ammonium molybdate and incubated at 95°C for 90 min in water bath. The absorbance was measured at 695 nm, using ascorbic acid as a standard.

For C, H, N analysis, thalli of parent and putative hybrids were shed dried at a constant temperature of 35 ± 1°C until constant weight was achieved. 100 mg of the dried biomass was taken and carbon, hydrogen and nitrogen contents were determined using CHN analyser (Vario micro cube, Elementar, Germany).

### Statistical analyses

All the values for daily growth rate, carbohydrate, lipids, protein, cellulose, Fas and TAC for hybrids and their parents are reported as means ± SD (*n* = 2). Further the significant differences among the mean values were evaluated by Tukey's HSD test after performing One-Way analysis of variance (ANOVA) using statistical software, JMP 11.0 (trial version), values significant at *p* < 0.05. The distribution of hybrids and the fusion partners based on their fatty acid composition was analyzed by Principal component analysis (PCA). For PCA analysis data was mean centered and accomplished using Unscrambler 9.8 software package (CAMO AS, Trondheim, Norway).

## Results

**Figure 1:**
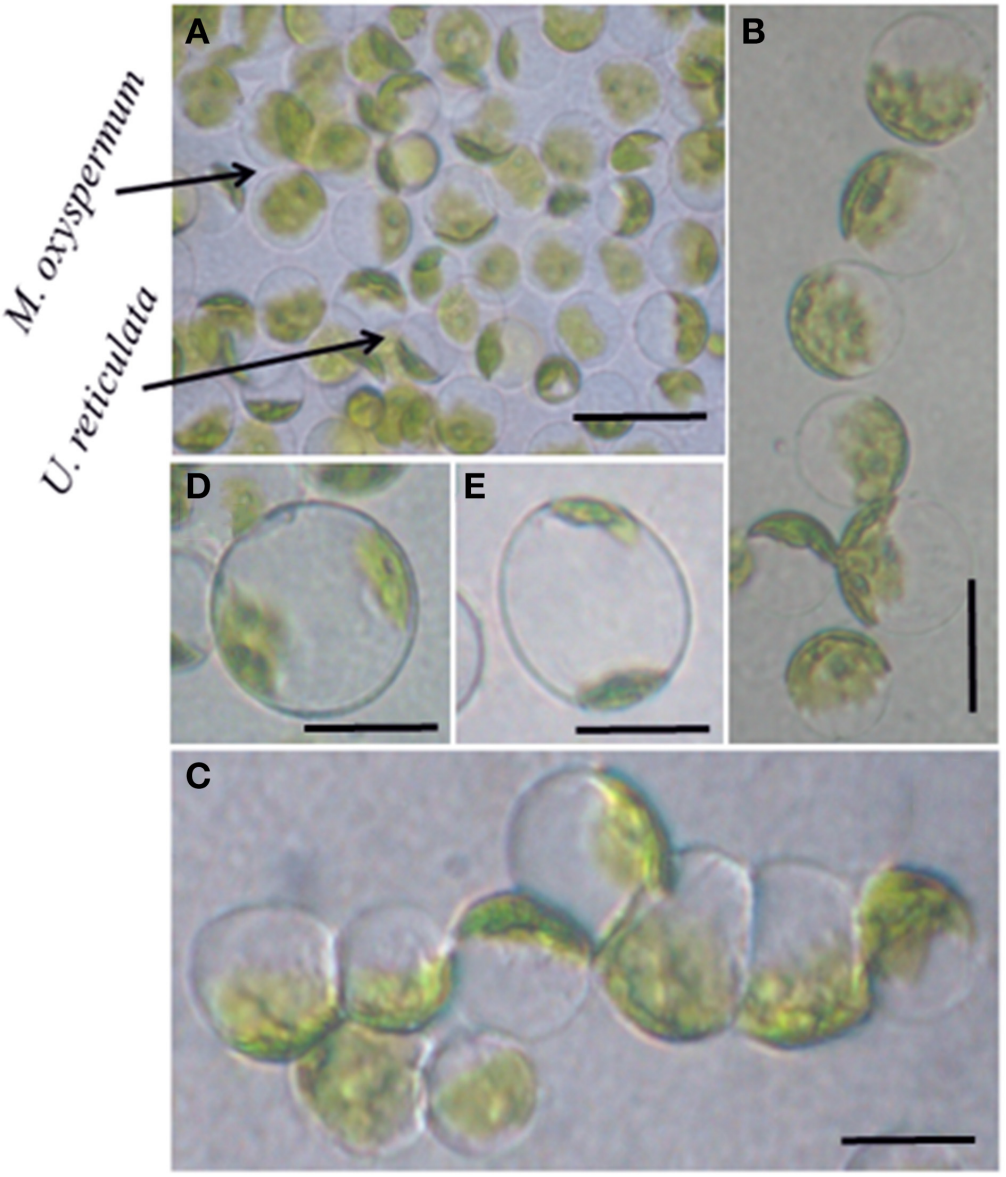
**Protoplast fusion between the selected species *M*. *oxyspermum* and *U*. *reticulata***. **(A)** Mixture of protoplasts isolated from the selected species, **(B,C)** protoplasts alignment and pearl-chain formation after applying AC pulse, **(D,E)** protoplast fusion after applying DC pulse showing two chloroplasts in a single cell. Scale bar = 50 μm.

**Figure 2:**
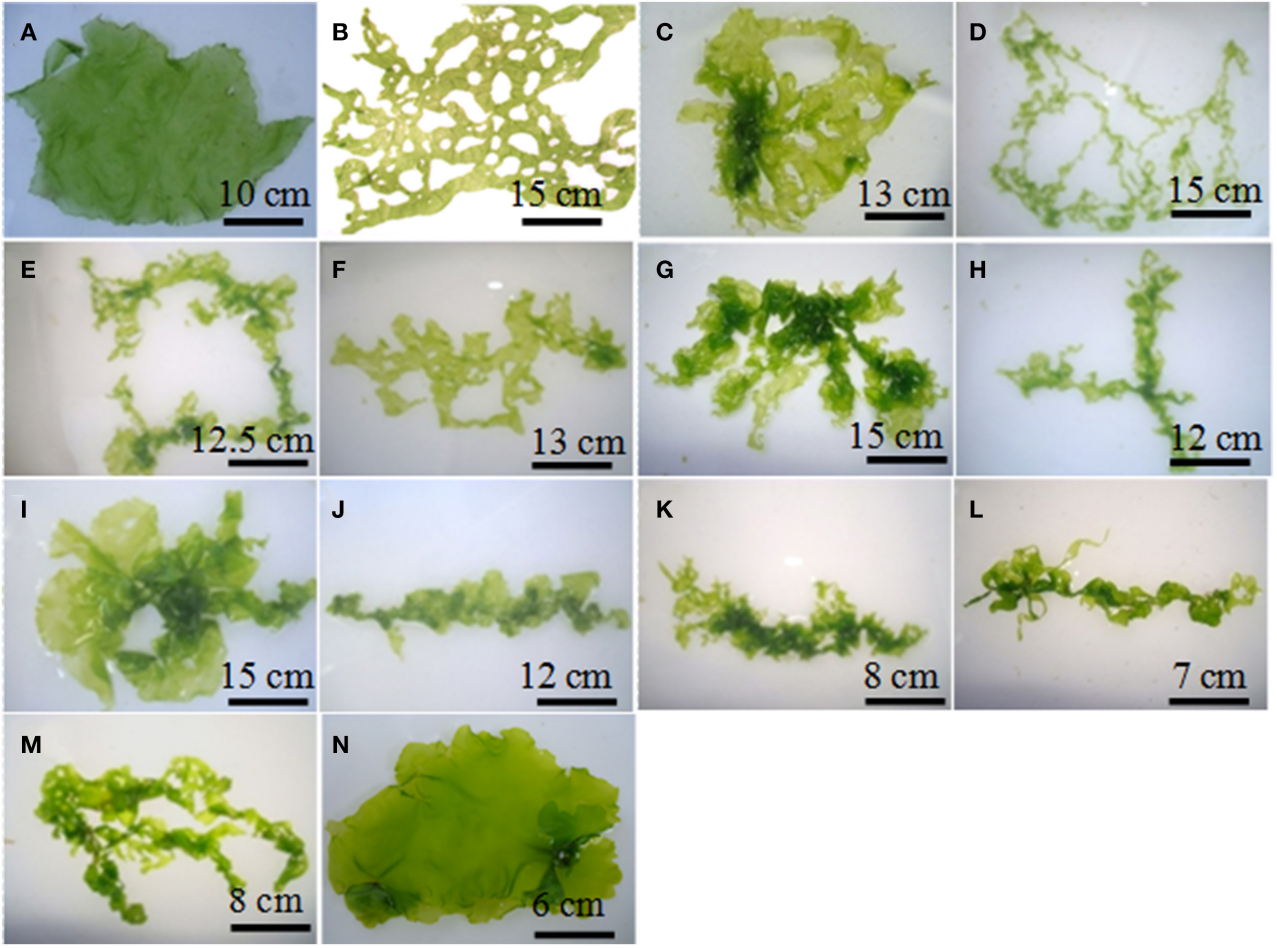
**The morphology of fusion partners and putative hybrids generated after protoplast fusion**. **(A)** *M*. *oxyspermum* and **(B)** *U*. *reticulata*, **(C–N)** putative hybrids. The age of the hybrids represented in the figure is 4 months.

The protoplasts isolated from *M. oxyspermum* and *U*. *reticulata* were fused using the electrofusion method under an electric field (Figure 1A). The optimization of electrofusion conditions showed that the application of an AC voltage of 40 V for 25–30 s yielded alignment of about 70–80% of protoplasts into short chains of 3–7 protoplasts (Figure 1B). Subsequently, application of a single higher-voltage DC pulse of 300 V for 20 μs duration induced about 4–6% fusions in pre-aligned protoplasts (Figure 1C). The fusion products were identified based on the larger size of the cell and presence of two morphologically distinct chloroplasts in a fused cell compared to unfused protoplasts (Figures 1D,E). On subsequent culturing of fused cells, it was observed that many cells were unable to regenerate even the cell wall. A total of 22 fusion products showed cell wall formation in 3 days and underwent repeated cell division and differentiation giving rise to plantlets after 4 weeks of culture. The regenerated plantlets were then transferred to aerated culture flasks where regenerants attained full thallus morphology after 4–6 weeks of culture. Of the 22 plantlets screened, a total of 12 germlings were finally selected as putative hybrids based on their distinct thallus morphology compared to both fusion partners. The thallus morphology of putative hybrids and the fusion partners is shown in Figure 2. The thallus architecture of regenerated putative hybrids was distinct from that of both fusion partners. Of the fusion partners, *M. oxyspermum* had a flat monostromatic sheet-like thallus while *U*. *reticulata* had a typical distromatic, reticulated thallus. However, all the regenerated putative hybrids were distromatic but diverged from wild type morphology of *U. reticulata* and were distinct from each other (Figure 2).

### Ploidy analysis using confocal microscopy

**Figure 3:**
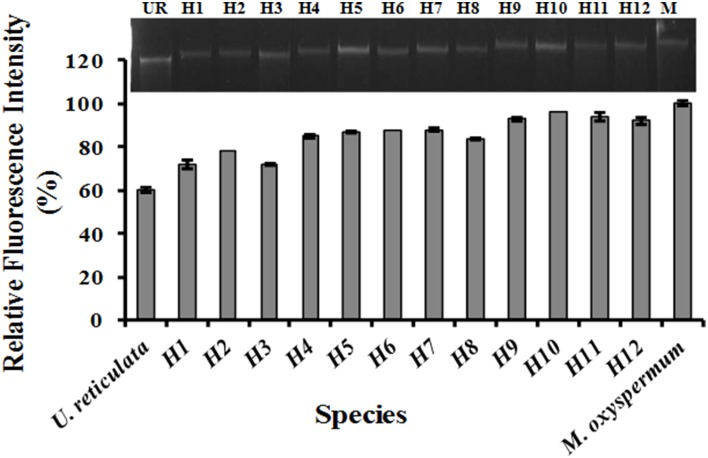
**Relative fluorescence intensity of the regenerated hybrids and the fusion partners determined from confocal microscopy**. The gel image above the graph showed the migration of total genomic DNA of hybrids and fusion partners. UR, *U*. *reticulata*; M, *M*. *oxyspermum;* H1–12, Putative hybrids.

Following treatment with DAPI, blue fluorescence was observed in stained nuclei. Both the nuclei and chloroplast were clearly distinguished when the slides were observed in bright field with superposition of the nuclear fluorescence and chloroplast autofluorescence (Supplementary Figure 1). The fusion partners and the regenerated putative hybrids showed clear differences in nuclear fluorescence intensity. The relative difference in the fluorescence intensity among the fusion partners and the putative hybrids was used as a measure for analysing the ploidy. Among the fusion partners, *M. oxyspermum* showed the highest nuclear fluorescence intensity and the nuclear fluorescence intensity for rest of the genotypes was estimated relative to it. The fluorescence intensity shown by other fusion partner, i.e., *U*. *reticulata* was 60% of *M. oxyspermum*. The putative hybrids showed fluorescence intensity ranging between 72 and 92% (Figure 3). In addition, the total genomic DNA showed differences in migration rate when run on an agarose gel, revealing variation in genome sizes (Figure 3). These results showed that the regenerated hybrids contained DNA content (ploidy) greater than that of the fusion partner *U*. *reticulata* but less than that of the other partner *M. oxyspermum* suggesting that they are asymmetric hybrids.

### Molecular analysis of putative hybrids

The molecular characterization of regenerated hybrids was carried out using ISSR, AFLP and MSAP analysis.

### ISSR profiling

The hybrid nature of the regenerants was further confirmed by ISSR profiling. A total of 8 primers gave highly reproducible polymorphic bands in the somatic hybrids and their fusion partners. A total of 428 bands were scored of which 348 were polymorphic among the fusion partners. The ISSR profile revealed the species-specific bands of both fusion partners in the somatic hybrids, which confirmed their hybrid nature. The ISSR profile is shown in Supplementary Figures 2A,B, in which transfer of the fusion partner -specific band in the regenerated hybrids is shown. The ISSR analysis of hybrids (1–12) revealed a genetic similarity of 62, 67, 58, 62, 56, 64, 68, 68, 46, 62, 54, 56% respectively with *U*. *reticulata* and 42, 44, 52, 46, 58, 56, 42, 44, 63, 58, 68, 72% respectively with *M*. *oxyspermum*. These findings confirmed the introgression of genomes of both the fusion partners into the regenerated hybrids.

### AFLP analysis

**Figure 4:**
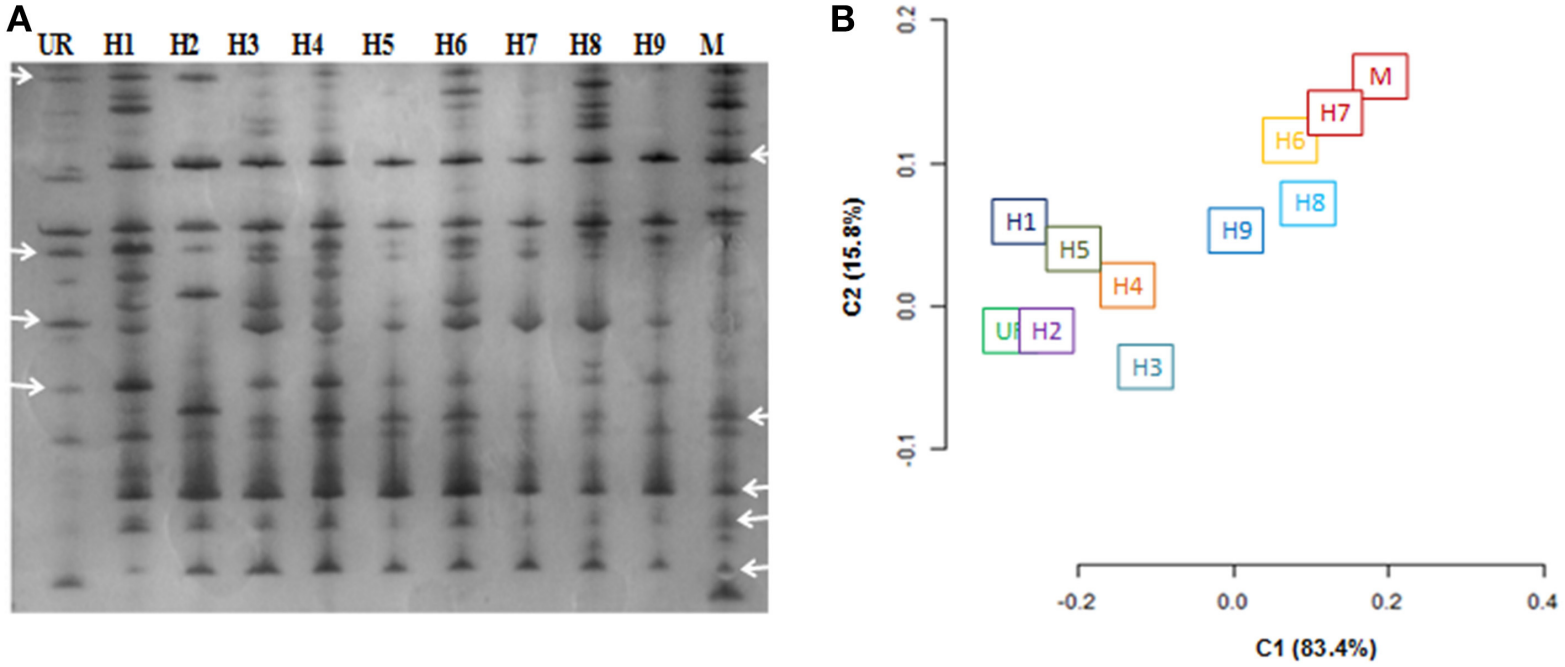
**Characterization of regenerated hybrids based on AFLP analysis. (A)** AFLP gel wherein arrowheads showed the bands shared by hybrids with either of the fusion partner **(B)** AFLP based clustering of regenerated hybrids and fusion partners. % represents the explained variance at PC1 and PC2. UR, *U. reticulata*; M, *M. oxyspermum*; H1–9, Hybrids.

AFLP profiles for three putative hybrids, i.e., H3, H7, H12 were not obtained in a reproducible manner so were excluded from subsequent studies. As a result of the AFLP analysis, a total of 324 bands were scored. The AFLP profile of the two fusion partners showed distinct band profiles, with 286 polymorphic bands (Figure 4A). The regenerated hybrids showed bands common to both the partners. The band-sharing frequency analysis revealed a higher match for hybrids 1–5 with *U. reticulata*, while the other hybrids (6–9) were more similar to *M. oxyspermum*. In addition to sharing common bands with fusion partners, a few additional bands that were not present in either fusion partner were also observed in hybrids. However, the frequency of such additional bands was less than 1% of the total bands. The distribution plot based on band-sharing is shown in Figure 4B. The principal co-ordinate distribution clearly showed that both fusion partners were at the extreme ends and the regenerated hybrids remained between the partners with proximity to either parent according to their band-sharing frequency. The AFLP analysis coincides well with the inferences made from the ISSR and ploidy analyses. Combining all the results, i.e., morphology, ploidy analysis, and molecular characterization, has confirmed the genetic introgressions in the regenerated hybrids.

### MSAP analysis

A total of 324 methylation loci were scored for MSAP analysis. The loci were classified as unmethylated, hemi-methylated at the external cytosine, fully methylated at the internal cytosine, and fully methylated at the external cytosine or both the internal and external cytosines (Supplementary Figures 3A,B). Both the parent plants showed distinct methylation profiles. *M. oxyspermum* showed significantly higher DNA unmethylation (38%) and hemimethylation (24%) rates compared to *U*. *reticulata* with corresponding values of 19 and 11% respectively. The methylation frequency of the internal cytosine and full methylation estimated for *U*. *reticulata* (39 and 31% respectively) was significantly higher than that of *M. oxyspermum* (26% and 12%). The frequency of total methylation was 88% in *M. oxyspermum* and 69% in *U*. *reticulata*. The fusion partners exhibited markedly heterogeneous DNA methylation patterns because of high degrees of methylation polymorphism, as 76.24%. Comparison of DNA methylation patterns with those of the regenerated hybrids revealed no large-scale alterations in DNA methylation compared to the fusion partners methylation profile. An increase in total methylation was observed for all the hybrids in comparison to *U. reticulata* while total methylation of the hybrids was lower than in *M. oxyspermum*. The increase in total methylation frequency was due to random increases in either of the methylated loci in different genotype (Table 1).

**Table 1:**
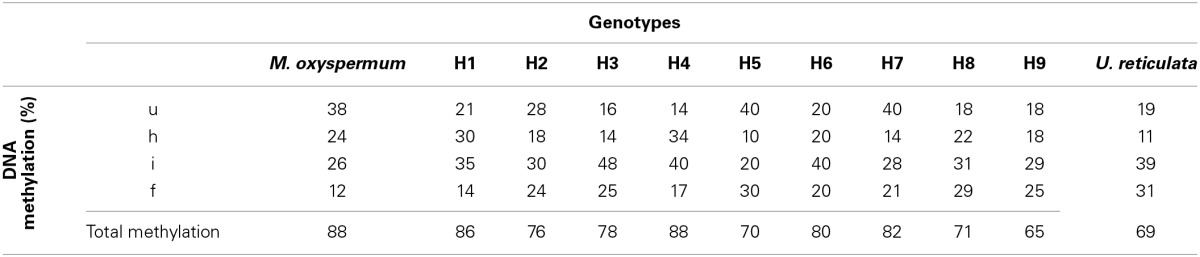
**Comparison of DNA methylation levels between fusion partners *M. oxyspermum* and *U*. *reticulata* and the somatic hybrids determined by methylation-sensitive amplified polymorphism (MSAP) assay**.

### Improvement in functional traits of regenerated hybrids

**Figure 5:**
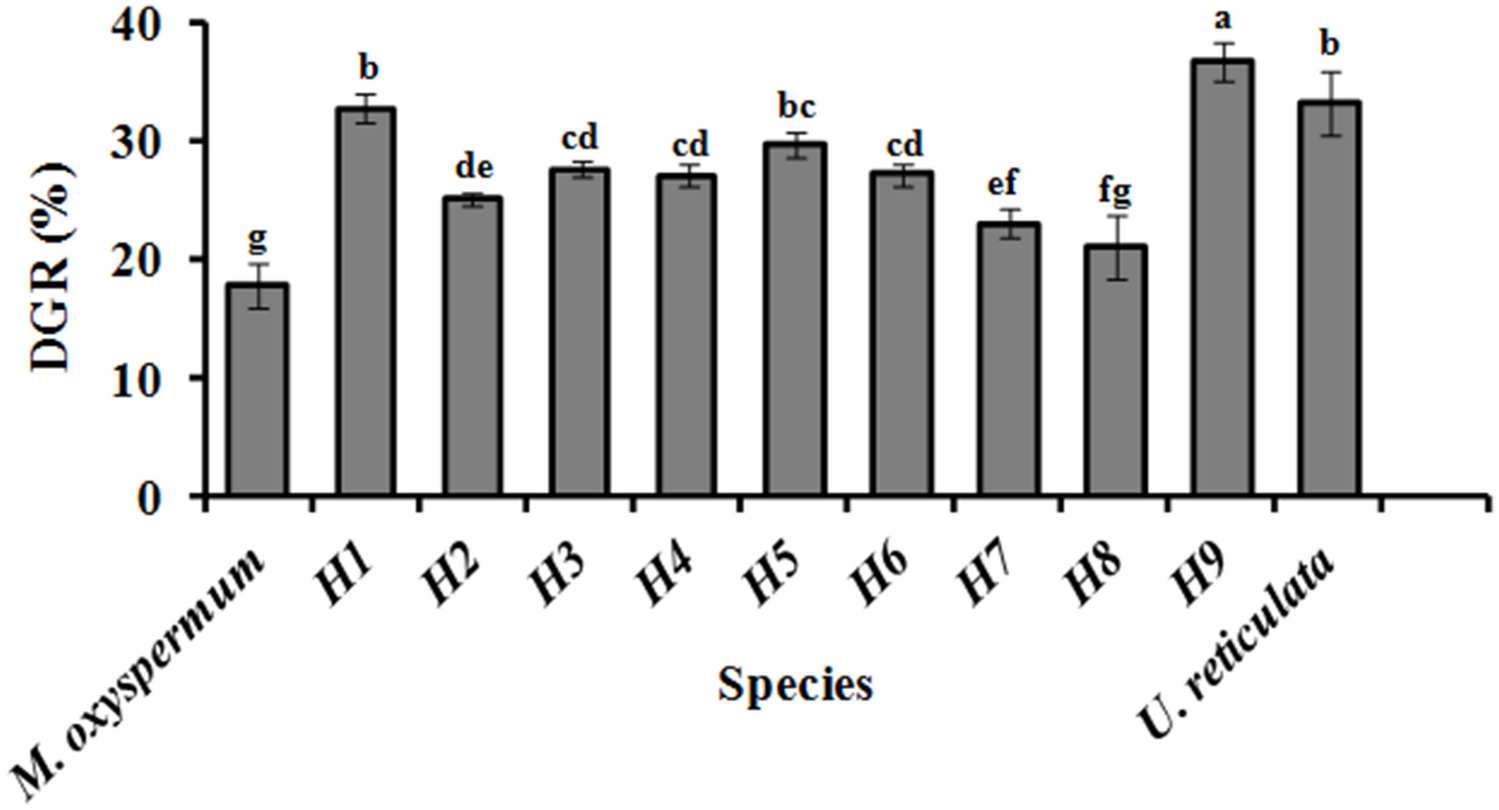
**Growth rate (DGR %) of the regenerated hybrids and the fusion partners**. H1–9, hybrids. Bars with different letters represent means significantly different from each other at *p* < 0.05.

Growth, fatty acids and proximate composition were analyzed for the regenerated hybrids and were compared with the fusion partners' traits. The growth rate analysis revealed significant differences between the DGR (%) of both the fusion partners. *U. reticulata* showed DGR of 33.2 ± 2.6% which is about 2-fold higher than that of *M. oxyspermum* (17.8 ± 1.77%), (*p* < 0.05). Among all the hybrids generated, Hybrid 9 showed DGR% of 36.7 ± 1.55% which is the highest among all the hybrids as well as greater than the fusion partner *U*. *reticulata* (*p* < 0.05). Other hybrids showed DGR higher than the mid-parent value, i.e., 25.5 ± 2.2% except for hybrid 2, 7, and 8 (*p* < 0.05) (Figure 5).

**Figure 6:**
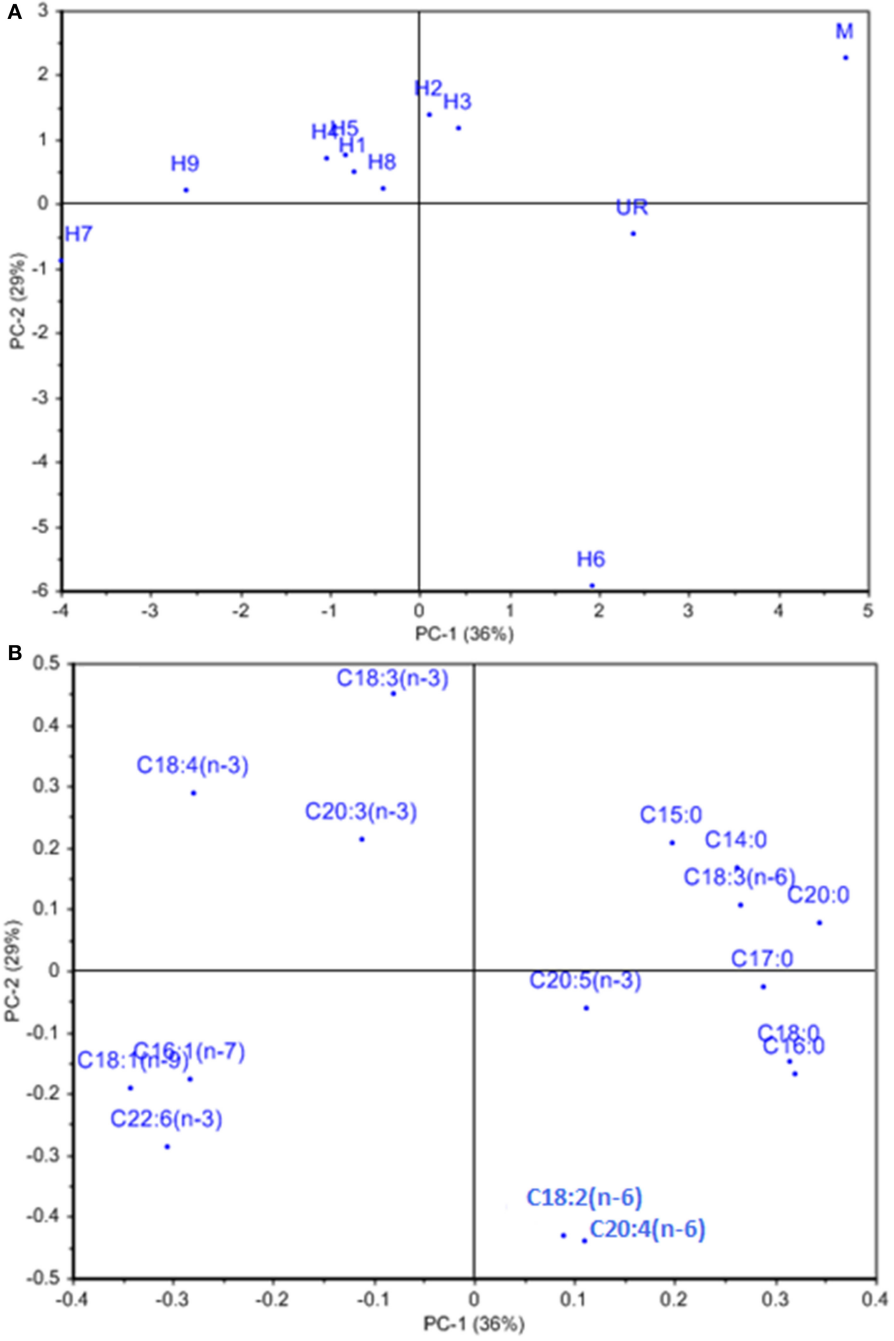
**(A)** Loading and **(B)** score plot of hybrids and the fusion partners obtained from PCA analysis of fatty acid data matrix. % represents the explained variance at PC1 and PC2.

The other characteristics investigated, including proximate composition of regenerated hybrids and the fusion partners are summarized in Table 2. The total carbohydrate content was 51.11 ± 2.2% for *U*. *reticulata* and 20.85 ± 1.12% for *M*. *oxyspermum* and ranged between 36.8 ± 1.8% and 52.5 ± 2.2% in the hybrids. Hybrid 9 had a total carbohydrate content higher than the fusion partners (thus a superior trait), while all other hybrids had a carbohydrate content greater than that of the average of both the fusion partners (32.5 ± 2.2%) (*p* < 0.05). The cellulose content was higher in *U*. *reticulata* (11 ± 1.75%) in comparison to *M*. *oxyspermum* (6.5 ± 0.22%) and the mid-parent value for this trait was 8.75 ± 0.98%. The cellulose content in the hybrids ranged between 6.1 ± 1.2% and 14.2 ± 2.2%. Similarly to the total carbohydrate content, hybrid 9 showed cellulose content higher than that of the fusion partners, indicating a superior trait (*p* < 0.05). The total protein content was higher in *M*. *oxyspermum* (11.75 ± 2.6%) than *U*. *reticulata* (6.8 ± 2.12%) and the mid-parent value for this trait was 9.27 ± 2.36%. The content of total protein in the fusion partner was in reverse order in comparison to the content of carbohydrate. The protein content of the hybrids was found to be in the range from 7.1 ± 1.5% to 9.2 ± 1.8%. The protein content of hybrid 1 was higher than the mid-parent value while others had lower protein content than the mid-parent value but higher than the parent having this as the inferior trait (*U*. *reticulata*) (*p* < 0.05). The total antioxidant capacity (TAC) was estimated as 0.64 ± 0.08 (mg AAE/g extract) and 0.44 ± 0.1 (mg AAE/g extract) for *M*. *oxyspermum* and *U*. *reticulata* respectively while the mid-parent value for this trait was 0.54 ± 0.09 (mg AAE/g extract). The estimated TAC in the hybrids was ranged from 0.33 ± 0.16 (mg AAE/g extract) to 0.72 ± 0.92 (mg AAE/g extract). Among the hybrids, hybrid 9 showed a TAC (0.72 ± 0.92 mg AAE/g extract) higher than that of the fusion partner with higher trait value (*p* < 0.05). The other hybrids, hybrid 2, 3, 7, and 8 showed a TAC value better than *U*. *reticulata* as well as higher or equal to the mid-parent value, while hybrid 1, 4, and 6 showed a TAC less than the mid-parent value but similar to *U*. *reticulata* (*p* < 0.05). The fatty acid (FA) compositions for all the genotypes are shown in Table 3. PCA of the FA data matrix provided a global statistical distinction between the fusion partners and the hybrids (Figures 6A,B). The FA profile investigated for the genotypes explained a total variation of 65% (PC1-39% and PC2-26%) revealing a distinct distribution of hybrids and the fusion partners. Both the fusion partners and the generated hybrids showed characteristic fatty acid profile with the richness of ω 3 and content of C18 PUFAs > C20 PUFAs. The PUFA content varied from 37 to 55% among the fusion partners and the hybrids generated. However, the sum of the content of arachidonic acid (C20:4 n-6) and eicosapentaenioc acid (C20:5 n-3) accounts for only ≤1.4% among the genotypes studied. The total saturated and unsaturated fatty acid content was analogous in the fusion partners; the former was higher in *M*. *oxyspermum* while the latter was higher in *U*. *reticulata*. The FA profile revealed an increase in unsaturation (1.1–1.6-fold) in nearly all the hybrids, (the only exception was hybrid 6), and a decrease in saturation for all the hybrids compared to the fusion partners. The increase in unsaturation was due to an increase in the contents of both PUFAs by 1.2–1.5-fold, mainly C18 PUFAs and MUFAs by 1.1–3.7-fold (except hybrid 3 and 5). The content of C18 PUFAs in the hybrids was higher than in both the fusion partners (1.1–1.5-fold) with the only exception being hybrid 6 that exhibited a different FA profile from rest of the hybrids. Interestingly, the FAs profile revealed that all the hybrids except hybrid 6 have an elevated PUFA content (including n-3 PUFAs), compared to the average of both the fusion partners (Table 3). Hybrid 6 had a higher saturated FA content (SFA/UFA = 1.1), mainly due to higher stearic acid (C18:0), and the lowest PUFA due to a decrease in C18 PUFA content, especially C18:3 (n-3) and C18:4 (n-3). Despite the decrease in total PUFAs, the content of C20 PUFAs increased in hybrid 6 by 1.9–3.1-fold due to increase in arachidonic acid content (C20:4, n-6). The other hybrids showed a predominance of palmitoleic (C16:1, n-7) and oleic acid (C18:1, n-9) among MUFAs. The content of oleic acid was 1.1–5.2-fold higher in all the hybrids compared to fusion partners (*p* < 0.05). The content of two essential FAs, ALA (C18:3, n-3) and LA (C18:2, n-6) differed among the fusion partners: the former was higher in *M*. *oxyspermum* while the latter was higher in *U*. *reticulata*. Interestingly the hybrids showed ALA content similar to *M*. *oxyspermum*, except for hybrids 6 and 7, while LA content was similar to *U*. *reticulata* except for hybrids 3 and 4 (*p* < 0.05). The content of palmitoleic acid (C16:1) was higher in hybrids 4, 7, and 9 while others showed an increase in oleic acid (C18:1, n-9) content with a 1.1–4.5-fold increase compared to *M. oxyspermum* and a 1.1–2.6-fold decrease compared to the content in *U. reticulata*. Among MUFAs, both heptadecenoic acid (C17:1, n-7) and oleic acid (C18:1, n-9) showed the traits of heterosis (superior hybrids compared to both the fusion partners) while C16:1 (n-9) showed heterosis in three hybrids (4, 6, and 9) while three hybrids (1, 2, and 8) had the traits of mid-parent heterosis. Similarly, among PUFAs, stearidonic acid (C18:4, n-3) (except hybrid 6) and docosahexanoic acid (C22:6, n-3) showed higher contents than both the fusion partners. All these contents showed improvement in traits, therefore indicating the potential of this technique to develop elite germplasm. All these results summarized that hybrid 9 showed improved functional traits for growth, carbohydrate and cellulose content, anti-oxidative potential and fatty acid profile compared to the fusion partners.

**Table 2:** **Comparison of the proximate composition of regenerated hybrids and the fusion partners**.

**Components (% on dry wt basis)**	***M. oxyspermum***	**Hybrid 1**	**Hybrid 2**	**Hybrid 3**	**Hybrid 4**	**Hybrid 5**	**Hybrid 6**	**Hybrid 7**	**Hybrid 8**	**Hybrid 9**	***U*. *reticulata***
Carbohydrate	20.85±0.91^d^	37.87±1.62^bc^	44.55±1.90^ab^	41.2±3.53^b^	42.2±1.97^b^	37.1±2.40^bc^	38.5±1.69^bc^	36.8±1.71^bc^	37.1±1.83^bc^	32.5±1.8^c^	51.11±0.43^a^
Mid-parent value						35.98±0.67					
Protein	11.75±2.68^a^	9.2±1.55^ab^	8.85±2.33^ab^	8.8±2.26^ab^	7.1±1.13^b^	8.4±0.98^ab^	7.1±0.56^b^	7.1±1.71^b^	7.4±0.70^b^	7.8±0.84^b^	6.8±1.83^b^
Mid-parent value						9.27±2.25					
Lipid on Fwt basis	1.73±0.29a^b^	0.91±0.36^cd^	0.42±0.15^de^	0.49±0.07^de^	0.56±0.19^de^	0.43±0.26^de^	0.9±0.21^cde^	0.34±0.15^e^	0.54±0.14^de^	1.42±0.45^bc^	2.03±0.25^a^
Mid-parent value						1.88±0.27					
Cellulose	6.5±0.38d^e^	6.5±1.01^de^	8.2±1.97^bcde^	9.8±1.97^bc^	6.1±1.27^e^	9.4±0.98^bcd^	7.1±0.42^cde^	7.8±1.41^cde^	7.8±0.56^cde^	14.2±1.97^a^	11±1.69^b^
Mid-parent value						8.75±1.03					
TAC (mg AAE/g extract)	0.64±0.11^ab^	0.43±0.07^abc^	0.6±0.16^abc^	0.53±0.08^abc^	0.41±0.11^bc^	0.33±0.15^c^	0.48±0.19^abc^	0.58±0.08^abc^	0.55±0.21^abc^	0.72±0.05^a^	0.44±0.11^abc^
Mid-parent value						0.54±0.11					

*Values with different letters above represent means significantly different from each other at p < 0.05 (mean ± SD)*.

**Table 3:** **Fatty acid composition (given in % of total fatty acid methyl esters) of fusion partners and the regenerated hybrids (mean ± SD)**.

**FAs**	***M*. *oxyspermum***	**Hybrid 1**	**Hybrid 2**	**Hybrid 3**	**Hybrid 4**	**Hybrid 5**	**Hybrid 6**	**Hybrid 7**	**Hybrid 8**	**Hybrid 9**	***U*. *reticulata***
C12:0	1.15±0.05^a^	0.4±0.18^cd^	nd	0.4±0.11^cd^	0.2±0.02^de^	0.7±0.02^b^	nd	0.1±0.02^e^	nd	0.3±0.02^de^	0.6±0.07^bc^
C14:0	4.37±0.51^ab^	2.8±0.56^cd^	4.1±0.5^ab^	2.3±0.3^de^	3.2±0.28^cd^	1.6±0.28^bc^	1.8±0.24^ef^	0.7±0.26^fg^	4.8±1.4^a^	0.4±0.11^g^	3.8±0.56^abc^
C15:0	0.6±0.28^a^	0.4±0.28^ab^	0.6±0.28^a^	0.4±0.28^ab^	0.3±0.02^ab^	0.3±0.02^ab^	0.3±0.03^ab^	0.2±0.05^b^	0.2±0.03^b^	0.3±0.02^ab^	0.2±0.02^b^
C16:0	32.86±3.1^a^	29.7±2.8^ab^	26.3±0.7^bc^	26±1.7^bc^	26±0.3^bc^	30±1.7^ab^	33.4±1.8^a^	25.9±2.01^bc^	25.9±0.84^bc^	24.3±1.55^c^	33.9±3.01^a^
C17:0	0.52±0.04^a^	0.2±0.02^cd^	0.4±0.03^ab^	0.5±0.11^a^	0.4±0.07^a^	0.2±0.03^cd^	0.5±0.07^a^	0.1±0.01^d^	0.3±0.02^bc^	Nd	0.2±0.11^cd^
C18:0	14.87±0.77^a^	5.2±0.84^c^	4.3±0.7^c^	9.3±2.6^b^	5.4±0.56^c^	3.3±0.7^c^	14±0.28^a^	3.5±0.28^c^	4.8±0.84^c^	7.9±1.1^b^	7.9±0.98^b^
C20:0	0.65±0.14^a^	0.2±0.02^b^	0.1±0.02^b^	0.2±0.02^b^	0.2±0.03^b^	0.3±0.05^b^	0.2±0.16^b^	nd	0.1±0.05^b^	nd	0.7±0.3^a^
C22:0	1.53±0.02^abc^	1.7±0.14^ab^	1±0.14^c^	1.6±0.28^ab^	1.9±0.42^a^	1.6±0.28^ab^	1.9±0.28^a^	1.7±0.28^ab^	1.5±0.28^abc^	1.4±0.28^abc^	1.3±0.28^bc^
C24:0	1.85±0.35^a^	0.3±0.28^b^	0.2±0.02^b^	0.2±0.05^b^	0.2±0.02^b^	nd	0.5±0.35^b^	0.2±0.05^b^	0.1±0.02^b^	0.3±0.2^b^	nd
C16:1(n-7)	0.97±0.18^bc^	2.3±0.28^bcd^	2.2±0.7^bcd^	1.1±0.6^d^	3.1±0.56^ab^	1.7±0.84^cd^	2.6±0.28^bc^	4.4±0.7^a^	2.1±0.21^bcd^	3.4±0.56^d^	2.9±1.13^ab^
C17:1(n-7)	0.09±0.04^f^	0.3±0.03^de^	0.2±0.03^ef^	0.3±0.02^de^	0.6±0.11^ab^	0.3±0.05^de^	nd	0.7±0.11^a^	0.5±0.02^bc^	0.4±0.05^cd^	0.2±0.02^ef^
C18:1(n-9)	1.7±0.31^g^	5±0.21^bcd^	4.7±0.35^cde^	3.1±0.45^f^	4.7±0.56^cde^	4.4±0.3^de^	5.3±0.28^bc^	8.9±0.42^a^	4.6±0.28^cde^	5.7±0.3^b^	4±0.5^f^
C18:2(n-6)	4.04±0.21^c^	5.8±0.6^bc^	6.8±0.56^b^	3.8±0.56^c^	3.8±0.52^c^	5.8±1.4^bc^	16.6±2.12^a^	5.6±0.6^bc^	7.2±0.56^b^	5.5±0.42^bc^	7.3±0.7^b^
C18:3(n-6)	0.89±0.43^a^	0.4±0.11^b^	0.5±0.03^b^	0.4±0.02^b^	0.5±0.11^b^	0.5±0.11^b^	0.4±0.11^b^	0.5±0.07^b^	0.4±0.07^b^	0.4.03^b^	1±0.28^a^
C18:3(n-3)	21.33±2.78^a^	19±3.11^a^	21±2.96^a^	20.1±3.01^a^	19.3±3.1^a^	20.2±2.96^a^	10±2.4^b^	18.6±1.41^a^	19.1±2.4^a^	21±1.83^a^	17.3±1.55^a^
C18:4(n-3)	7.46±0.61^c^	20.4±2.12^a^	18.9±3.4^ab^	23±2.54^a^	23.3±1.83^a^	22.7±2.68^a^	0.7±0.17^d^	19.2±2.82^ab^	20.7±2.4^a^	22.1±1.41^a^	14.7±1.55^b^
C20:3(n-3)	1.73±0.12^a^	1±0.14^b^	0.6±0.28^bc^	0.6±0.14^bc^	1±0.07^b^	1±0.02^b^	0.2±0.01^cd^	1.7±0.6^a^	1±0.22^b^	1±0.26^b^	nd
C20:3(n-6)	0.09±0.01^c^	0.1±0.01^c^	Nd	nd	0.1±0.01^c^	Nd	0.6±0.2^b^	0.2±0.03^c^	nd	nd	0.9±0.07^a^
C20:4(n-6)	0.43±0.07^b^	0.5±0.05^b^	0.2±0.05^b^	0.7±0.28^b^	0.6±0.11^b^	0.3±0.16^b^	4.5±0.98^a^	0.6±0.03^b^	0.8±0.17^b^	0.6±0.11^b^	0.9±0.11^b^
C20:5(n-3)	0.73±0.14^bc^	0.2±0.05^c^	0.2±0.02^c^	0.2±0.04^c^	0.3±0.09^c^	0.3±0.09^c^	0.6±0.16^bc^	0.5±0.08^bc^	1.7±0.56^a^	0.3±0.14^c^	1±0.56^b^
C22:6(n-3)	1.2±0.56^e^	4.3±1.13^bcd^	3.4±0.7^cd^	3.9±1.13^cd^	4.8±0.98^abc^	4.7±0.70^abc^	6±0.84^ab^	6.4±0.98^a^	4.1±0.98^cd^	4.5±0.56^bc^	2.6±0.14^de^
∑SFA	58.4±2.33	40.9±3.67	37.1±1.71	40.8±2.61	37.9±1.41	37.9±2.73	52.5±4.64	32.6±1.45	37.8±2.83	35.0±2.65	48.8±2.61
∑UFA	41.6±2.25	59.3±4.24	62.8±4.63	59.2±5.41	62.0±6.64	62.0±4.67	47.4±2.63	67.3±3.48	62.1±5.69	64.9±4.48	51.8±2.59
∑MUFA	3.8±0.56	7.6±0.67	11.2±0.29	6.4±0.75	8.4±0.72	6.5±0.53	7.8±0.52	14.0±1.62	7.2±0.79	9.6±0.57	7.1±0.65
∑PUFA	37.9±2.73	51.7±4.33	51.6±3.24	52.7±3.46	53.6±2.48	55.5±4.83	39.6±149	53.3±3.72	55.0±3.69	55.3±1.53	44.8±1.53
∑C18 PUFA	33.7±3.82	45.6±2.25	47.2±2.83	47.3±2.61	46.9±2.58	49.2±4.49	27.7±1.67	43.8±3.68	47.4±2.76	48.9±1.48	40.3±3.48
∑C20 PUFA	3.0±0.28	1.8±0.56	1.0±0.24	1.5±0.54	1.9±0.54	1.5±0.68	5.9±0.54	3.0±0.73	35.0±2.45	1.9±0.72	1.9±0.63
∑n3 PUFA	32.4±3.36	44.9±2.35	44.0±2.29	47.8±2.63	48.6±2.45	48.9±3.78	17.5±1.48	46.4±3.52	46.6±2.72	48.9±1.51	35.6±1.52
∑n6 PUFA	5.4±0.45	6.8±0.37	7.6±0.23	5.0±0.57	5.0±0.38	6.6±0.59	22.1±1.63	6.9±0.63	8.4±0.57	6.4±0.43	9.2±0.48
n6/n3	0.2±0.03	0.2±0.41	0.2±0.11	0.1±0.07	0.1±0.02	0.1±0.07	1.3±0.68	0.1±0.01	0.2±0.06	0.1±0.03	0.3±0.11

*Values in the same row with different superscripts letters are significantly different at p < 0.05*.

*nd, not detectable*.

## Discussion

Plant selection and breeding techniques have been extensively applied for genetic improvements in agriculture crops, but remained underutilized for seaweeds despite their proven economic and commercial values. This study demonstrated the development of seaweed hybrid varieties with improved functional traits through protoplast fusion between species of the genus *Monostroma* and *Ulva*. *Monostroma* and *Ulva* species are well known for their nutritional values and both have characteristic growth and biochemical composition. The hybrids generated in this study showed heterozygous vigor (heterosis) displaying some traits superior to both the fusion partners. Earlier reports on the somatic hybridization of seaweeds have largely dealt with morphology and pigmentation, albeit showing a few improved traits such as growth (Reddy et al., 1992) and fatty acid composition (Reddy and Fujita, 1989) but lacked the evidence for the molecular level of recombination. Also, most of the studies reported the development of chimeric thalli after protoplast fusion indicating independent segregation of the two genomes without recombination. The most recent report of the development of a hybrid variety of the seaweed was that of Kito et al. (1998), between *Monostroma nitidum* and *Porphyra yezoensis*, which showed development of chimeric thalli with distict morphology as string- and bud-like thallus development, and confirmed genetic introgression based on RAPD marker analysis. This study also showed fatty acid profiles characteristic of both red and green seaweeds in regenerated hybrids. In the present study, evidence of recombination between the two genomes of the fusion partners in hybrid progeny was confirmed by independent DNA fingerprinting techniques (ISSR and AFLP) coupled with critical morphological observations of hybrids over fusion partners. In addition, for the first time provided additional insights into the transmission of epigenetic traits in hybrids based on the analysis of methylation sensitive loci. The shown morphological variations by hybrids might be a result of protoplast-differentiation under axenic condition. The previous studies have shown that the microflora associated with the seaweeds regulates growth and morphogenetic ability of seaweeds (Marshall et al., 2006; Singh et al., 2011). Wichard and Oertel (2010) showed that seaweed associated bacteria secrete morphogenetic factors that control the thallus morphogenesis in *Ulva*. Due to the lack of PCR analyses we cannot rule out, that the cultures presented in Figure 2 contain bacteria, which are, e.g., not cultivable. Therefore, present bacteria might still have an impact on the morphogenesis of the hybrids. Nevertheless, the molecular analysis of somatic hybrids carried out in this study showed the genome complement of both the fusion partners and thus confirms unambiguously the hybrid nature of the somatic hybrids.

The hybrids regenerated in this study were asymmetric since the ploidy content was not additive of both the genomes of the fusion partners. The previous study on protoplast fusion between *U. pertusa* and *E. prolifera* resulted in hybrids with no change in chromosome number (Reddy et al., 1992) whereas Kapraun (1989) reported parasexual fusion products of *Enteromorpha* with diploid and tetraploid levels. The occurrence of a non-additive chromosome count is a common phenomenon and reported in several studies of somatic hybridization in land plants (Chen et al., 2004; Minquin et al., 2008; Tu et al., 2009; Wang et al., 2011) but this study is the first in macroalgae. Partial introgression of chromosomes from one fusion partner to the other has been described in developing the monosomic lines of a wheat-rye combination (Fu et al., 2013). In the same study, elimination of chromosomes was also illustrated. The chromosome elimination was mainly attributed to differences in the cell cycle. The differences in the growth rates between fusion partners in this study could perhaps result in chromosome elimination in somatic hybrids due to variation in cell cycles. Addition and introgression of either full or part chromosomes of one parent partner into another has also been reported in hybrids of *Brassica rapa* (×) *Isatis indigotica* (Tu et al., 2009).

The varieties developed through protoplast fusion displayed either heterosis or mid-parent heterosis for the various traits investigated. With the advent of random genetic recombination, the expression of various functional traits was found to be variable among the somatic hybrids. The contents of carbohydrate and cellulose in the somatic hybrids were found to be higher than that of fusion partner *M*. *oxyspermum* (*p* < 0.05), while the contents of protein and total antioxidant potential were better than that of the other fusion partner *U*. *reticulata*, and the growth rate was improved in comparison to *M*. *oxypermum* with one hybrid having growth rate even higher than *U*. *reticulata*. Both the fusion partners and the generated hybrids showed characteristic fatty acid profile with the richness of ω 3 and content of C18 PUFAs > C20 PUFAs. This FA profile is in agreement with recent studies (Khotimchenko et al., 2002; Pereira et al., 2012; Kumari et al., 2013; Alsufyani et al., 2014). The PUFA content varied from 37 to 55% among the fusion partners and hybrids. However, the sum of the content of arachidonic acid (C20:4 n-6) and eicosapentaenioc acid (C20:5 n-3) accounts for only ≤1.4% among the genotypes studied. Recently, Alsufyani et al. (2014) reported polyunsaturated aldehydes (PUAs) production in *Ulva* due to wounding induced chemical transformation of PUFAs mainly the C20:4 n-6 and C20:5 n-3. The lower content of both these FAs in the present study might be due to the chemical transformation of these FAs into PUAs. However, the PUAs production is highly species dependent and reported to vary significantly among *Ulva* species (Alsufyani et al., 2014) and diatoms (Wichard et al., 2005). The fatty acid profile of the somatic hybrids revealed a mixed profile from both the fusion partners. The hybrids showed a decline in saturated fatty acid content with concomitant increase in unsaturated fatty acids. Among the unsaturated fatty acids, PUFAs contributed more to the increase in unsaturation. Interestingly, the content of two indispensable fatty acids LA and ALA, which cannot be synthesized by human body, were in opposing levels among the fusion partners. The former was higher in *U*. *reticulata* while latter was higher in *M*. *oxyspermum*. The hybrids showed both LA and ALA content from the fusion partner having this trait superior. This further confirms the genetic recombination in hybrids expressing the superior traits from the fusion partners. The content of DHA was found to be higher in all the hybrids in comparison to their fusion partners while the content of EPA was inferior in all the hybrids compared to the fusion partners. This could be due to the higher transcriptional activity of downstream elongase and saturase enzymes. The higher content of C18 PUFAs (LA, ALA, STA) is the characteristic feature of Chlorophycean species belonging to genus *Ulva* (Kumari et al., 2013). On the contrary, *Monostroma* showed a higher content of saturated fatty acid and lower content of PUFAs. The n6/n3 ratio for all the hybrids were recorded in the nutritionally beneficial range (0.1:1 to 1.3:1) and was improved compared to the fusion partners. The increase in n6/n3 ratio could further aid in decreasing low-density lipoproteins, cholesterol levels, preventing inflammatory, cardiovascular and nervous system disorders. Therefore, these hybrid varieties may be a better source of n3 PUFAs for the development of healthier food formulations. The PUFA/SFA ratio, which determines the nutritional value of lipids, is in accordance with the nutritional guidelines (≥0.4). Interestingly this ratio was found to be higher in hybrids in comparison to both the fusion partners.

Though heterosis has been known since Darwin, the underlying molecular mechanisms are little known (Miller et al., 2012; Hopkins, 2013). The current hypothesis is that the genetic input from each parental line serves to mask the deleterious alleles of the other, resulting in hybrid plants that are superior to either parent. It is presumed that changes in gene expression and regulatory networks are responsible for heterosis in hybrids (Birchler et al., 2003). Epigenetic regulation, as a change in DNA methylation, is gaining prominence nowadays as a means to establish the molecular basis for heterosis (Moghaddam et al., 2010). The changes in the cytosine methylation pattern are the result of elimination and/or introgression of donor chromatin from fusion partners (Cai et al., 2007). Also, a change in DNA methylation controls the expression of genes (Cai et al., 2007). Greaves et al. (2012) while analysing the DNA methylomes of two *Arabidopsis* ecotypes and their reciprocal hybrids revealed the altered methylomes in hybrids offspring in the form of non-additive methylation patterns in hybrids. This study also revealed the correlation between the DNA methylation changes in hybrids and the altered expression of the genes. In the present study an attempt was made to determine the transmission of polymorphic DNA methylation loci in the generated hybrids to highlight the possible underlying mechanism of heterosis. The findings revealed no Mendelian inheritance of the methylation profile but significant differences were observed among fusion partners as well as between the hybrids generated. Bergman and Mostoslavsky (1998) also reported that methylation patterns were not inherited as such from the parental genomes but established a new combination in each generation through a series of discrete regulated steps. It has been suggested that the loci that undergo methylation alteration are more likely to lead to expression of hybrid-specific genes and hence novel phenotypes (Zhang, 2008). A significant increase in the level of total DNA methylation was observed in hybrids in comparison to the fusion partners (Table 1). The fusion partners exhibited markedly heterogeneous DNA methylation patterns because of a high degree of methylation polymorphism (76.24%). However, comparison of fusion partner DNA methylation patterns with those of the regenerated hybrids revealed no large-scale alteration in DNA methylation. An increase in total methylation was observed for all the hybrids in comparison to *U. reticulata* while the same was lower than *M. oxyspermum*. The increase in total methylation frequency was due to a random increase in either of the methylated loci in different genotypes (Table 1). Similarly, no gross alteration in DNA methylation, histone modifications or transcript levels was observed for interspecific hybrids of *Arabidopsis thaliana* (Moghaddam et al., 2010). Hypermethylation is known to cause transcriptional inactivation of CG/CNG sites (Diequez et al., 1998) and has been highly associated with the formation of heterochromatin-mediated gene silencing (Klein and Coasta, 1997). The predominance of hypermethylation may lead to repression of spurious global transcription, in order to achieve efficient transcription or complete expression of desirable loci in hybrids. The observed significant difference in methylation profile is consistent with the hypothesis that the epigenetic changes are more pronounced when there is merger of two different genomes rather than by genome doubling (Madlung et al., 2002).

Among the hybrids generated, hybrid 9 showed the prevalence of superior traits over the fusion partners. Also, the hybrid 9 showed the biomass characteristics equivalent to most *Ulva* species with an additional complementation of fatty acid profile of *M*. *oxyspermum*. Moreover, the growth rate shown by hybrid 9 is superior to the growth rate of most *Ulva* species (Bruhn et al., 2011; Trivedi et al., 2013). Hybrid 9 showed the inheritance of complementary characteristics of both the fusion partners for example the higher growth rate from *U*. *reticulata* and nutritionally important FAs from *M*. *oxyspermum*. In addition to these traits, hybrid 9 showed cellulose content superior to the fusion partners. Hybrid 9 with nutritional characteristics of *M*. *oxyspermum* and ~double the growth rate of *M*. *oxyspermum* is of interest for cultivation. The vegetative propagation of such biomass offers the most important advantage of the reduction in harvest time which in turn may increase the total crop productivity and better economic gains. Moreover, the development of biomass with higher cellulose content along with better growth and nutritional richness is sought after for development of biorefinery for food, fuel and commodity products.

In conclusion, the present study demonstrates successful protoplast fusion and regeneration of somatic hybrids with improved traits of agronomical importance over their fusion partners. The genetic relatedness as well as distinctness of hybrids compared to fusion partners was confirmed by cytological and molecular evidences. The traits transferred to hybrids include the nutritional richness of *M. oxyspermum* and higher growth and cellulose content of *U. reticulata*. The inheritance of epigenetic variations as a change in methylation enabled us to understand the possible underlying mechanism pertaining to activation or expression of gene machinery, possibly explaining the observed heterosis of hybrids. Such hybrid material is of particular interest for genome-wide analysis studies to identify and characterize the background genetic loci regulating the functional traits. The developed somatic hybrids with improved nutritional value, growth rate and cellulose content unequivocally demonstrate the significance of the protoplast fusion technique for developing improved variants of economically important marine macroalgae.

## Conflict of interest statement

The authors declare that the research was conducted in the absence of any commercial or financial relationships that could be construed as a potential conflict of interest.
