# Supplementary material for: Development and Characterization of Somatic Hybrids of Ulva reticulata Forsskål (×) Monostroma oxyspermum (Kutz.)Doty
Source: Front Plant Sci. 2015 Jan 29;6:3. doi: 10.3389/fpls.2015.00003 (PMC4310296; doi:10.3389/fpls.2015.00003)
Supplement: Supplementary file 1 [file DataSheet1.DOCX]

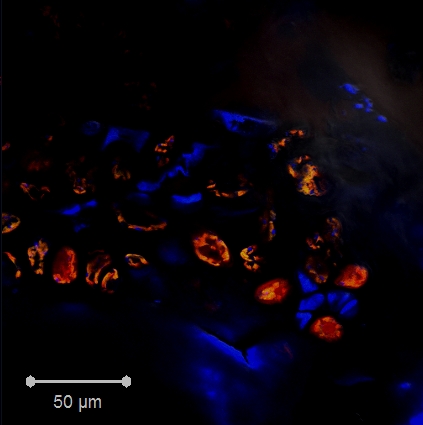


**Supplementary Figure 1|** Confocal microscopy based bright-field image of seaweed thalli with superposition of the nuclear fluorescence (blue) and chloroplast autofluorescence (red) for determination of ploidy. The cells in the image appear to be in division state.

A

B

**Supplementary Figure 2|** Characterization of regenerated hybrids by ISSR analysis. Arrow head showed the bands shared with either of the fusion partner.

M: Molecular weight marker (1.2 Kb); Mon: *M*. *oxyspermum*; H1-12: hybrids; UR: *U*. *reticulata*

A

B

**Supplementary Figure 3|** (A) Methylation sensitive amplification polymorphism profile for fusion partners and regenerated putative hybrids, (B) their cluster distribution based on band sharing. M: *M. oxyspermum*, UR: *U. reticulata* and H1-9: Hybrids.

**Supplementary Table 1|** Primer sequences for ISSR analysis.

| ISSR Primer sequence |
| --- |
| (CA)_6_ GG |
| (CA)_6_ AC |
| (CA)_6_ GT |
| (GA)_6_ GG |
| (GA)_6_ CC |
| (GT)_6_ CC |
| (AG)_8_ T |
| (AG)_8_ C |
| (CA)_6_ AG |
| (AG)_8_ C |
| (AC)_8_ T |
| (AC)_8_ G |
| (GATA)_4_ |
| (GACA)_4_ |
| (CA)_6_ G |
| (AG)_8_ GT |
| (AG)_8_ AT |
| (AG)_8_ GC |
| (AG)_8_ AC |
| (GATA)_4_ GG |

**Supplementary Table 2|** Sequences of adapters in methylation sensitive amplification polymorphism assay.

| **Adapter name** | **Sequence** |
| --- | --- |
| *Eco*RI-adapterI | 5’CTCGTAGACTGCGTACC 3’ |
| *Eco*RI-adapterII | 5’ AATTGGTACGCAGTC 3’ |
| *Hpa*II/*MspI*-adapterI | 5’ GACGATGAGTCTCGAT 3’ |
| *Hpa*II/*Msp*I-adapterII | 5’ CGATCGAGACTCAT 3’ |

**Supplementary Table 3|** Pre-amplification primers complementary to the EcoRI and HpaII/MspI adapters with one additional selective nucleotide at the 3’ end.

| **Adapter name** | **Sequence** |
| --- | --- |
| *Eco*RI | 5’ GACTGCGTACCAATTCA 3’ |
| *Hpa*II/*Msp*I | 5’ ATGAGTCTCGATCGGA 3’ |

**Supplementary Table 4|** Secondary selective primer combinations complementary to the *EcoR*I and *Hpa*II/*Msp*I adaptors with two or three selective nucleotides at the 3’ end.

| **Primers** | **Sequence** |
| --- | --- |
| *Hpa*II/*Msp*I-AAT | 5’ ATGAGTCTCGATCGGAAT 3’ |
| *Hpa*II/*Msp*I-ATC | 5’ ATGAGTCTCGATCGGATC 3’ |
| *Hpa*II/*Msp*I-ACT | 5’ ATGAGTCTCGATCGGACT 3’ |
| *Eco*RI -AC | 5' GACTGCGTACCAATTCAC 3’ |
| *Eco*RI -AA | 5' GACTGCGTACCAATTCAA 3’ |
| *Eco*RI -AG | 5' GACTGCGTACCAATTCAG 3’ |
| *Eco*RI -AT | 5' GACTGCGTACCAATTCAT 3’ |
